# A Simple Route to Produce Highly Efficient Porous Carbons Recycled from Tea Waste for High-Performance Symmetric Supercapacitor Electrodes

**DOI:** 10.3390/molecules27030791

**Published:** 2022-01-25

**Authors:** Xiaoxiao Qu, Weiwei Kang, Changwei Lai, Chuanxiang Zhang, Suck Won Hong

**Affiliations:** 1 Department of Cogno-Mechatronics Engineering, Pusan National University, Busan 46241, Korea; m18339169838@163.com; 2 College of Chemistry and Chemical Engineering, Henan Polytechnic University, Jiaozuo 454000, China; weiweikangwell@163.com; 3 School of Materials Science and Engineering, Anyang Institute of Technology, Anyang 455000, China; laichangwei0229@163.com

**Keywords:** tea waste, hydrothermal treatment, porous carbon, supercapacitor

## Abstract

High-performance porous carbons derived from tea waste were prepared by hydrothermal treatment, combined together with KOH activation. The heat-treatment-processed materials possess an abundant hierarchical structure, with a large specific surface of 2235 m^2^ g^−1^ and wetting-complemental hydrophilicity for electrolytes. In a two-electrode system, the porous carbon electrodes’ built-in supercapacitor exhibited a high specific capacitance of 256 F g^−1^ at 0.05 A g^−1^, an excellent capacitance retention of 95.4% after 10,000 cycles, and a low leakage current of 0.014 mA. In our work, the collective results present that the precursor crafted from the tea waste can be a promising strategy to prepare valuable electrodes for high-performance supercapacitors, which offers a practical strategy to recycle biowastes into manufactured materials in energy storage applications.

## 1. Introduction

Supercapacitors, including electric double-layer capacitors (EDLCs) and pseudocapacitors, have garnered continuous attention as an efficient energy storage system due to their higher power density, superior rate performance, longer cycle life, and a wider range of applications, compared with other energy storage systems [1,2,3,4,5,6,7,8]. In the material systems, electrodes play an important role in determining electrochemical performance [9,10,11,12,13]. Activated carbons (ACs) have been used as a conventional material for EDLC electrodes because of their intrinsic characteristic properties, such as high specific surface area, developed pore structure, and suitable surface chemical state [14,15,16]. Over the past years, much attention has been focused on transforming natural waste to worth-added ACs, such as carton [17], longan shell [18], sesame husk [19], lemon peel [20], walnut shells [21], lignocellulose [22], and palm [23]. As a result of the previous studies, it was found that there is great significance and research value in discovering low-cost and high-performance electrode materials by utilizing biowaste.

In China, the world’s largest tea-producing country [24], most of the tea waste is discarded as useless material, which leads to environmental pollution and resource waste. Therefore, there is a timely demand to search for an effective strategy for the efficient recycling of tea waste. In this context, it has been widely investigated to use tea waste as an electrode material associated with the high carbon content, pore structure, and large surface area, especially for supercapacitor applications. For example, the rod-like, porous carbon, with a specific surface area of 1610 m^2^ g^−1^, was synthesized through the pre-carbonization and simple KOH activation at 700 °C, using tea waste as a raw material [25]. The mesoporous graphitic carbon, prepared by KOH activation, from the green tea waste (i.e., carbon precursor) represented the specific capacitance of 162 F g^−1^ at 0.5 A g^−1^ [26]. The activated carbon derived from tea waste was fabricated by the ZnCl_2_ activation with a surface area of 1308 m^2^ g^−1^ and a total pore volume of 0.812 cm^3^ g^−1^ [27]. Additionally, the activated carbons produced from tea waste were prepared by chemical activation using K_2_CO_3_ and H_3_PO_4_ as activation agents, in which the highest specific capacitance of AC-K and AC-H electrodes are 203 F g^−1^ and 123 F g^−1^ at 1.5 mA cm^2^, respectively [28]. These previous reports indicated that tea waste could be utilized as excellent electrode materials for high-performance supercapacitors with a large surface area from the intrinsic porous structure.

Due to the advantages of low energy consumption and ease of processing [29,30,31], the hydrothermal method is currently one of the most attractive techniques for energy storage materials. Here, we developed a simple method to produce high-quality porous carbon materials derived from tea waste by using hydrothermal treatment, combined together with KOH activation, which is a well-established approach in the preparation of carbon-based electrode materials. For the recycling process of tea waste, we systematically surveyed the sufficient effect of the KOH agent in a delicately tailored manner to optimize the performance of the electrodes at a sufficient level of the porosity that entailed the highly beneficial structured surfaces for improved electrochemical performances in supercapacitors. In this study, we present an efficient and cost-effective approach that utilizes biowaste as a functional material for supercapacitor electrodes.

## 2. Materials and Methods

### 2.1. Materials

As a raw material, the typical tea waste (Xinyang Maojian, Xinyang, China) was ground into a powder (200 mesh). The KOH agent was purchased from Tianjin Damao Reagent Factory without any further purification.

### 2.2. Preparation of ACs

The two-step process, consisting of hydrothermal carbonization and chemical activation, was used to prepare the activated carbons. First, 40 g of tea waste was hydrothermally carbonized for 10 h at 180 °C with 400 mL of deionized water (DIW) in a 500 mL of stainless-steel autoclave, and the precipitates were gathered and dried. Next, the precursor and KOH with the mass ratios of 1:2, 1:3, and 1:4 were mixed with the DIW and dried for 10 h, and then the stock was transferred into a horizontal tube furnace at 800 °C for 1 h with N_2_ to achieve the activation. Finally, the as-prepared materials were soaked in 1 M HCl, washed with DIW, and dried to obtain the ACs. According to the different ratios of the precursor and KOH, the tea-waste-based activated carbon was named TAC, TAC2, TAC3, or TAC4, in which the TAC was the sample after the hydrothermal process without the KOH.

### 2.3. Characterization

The morphology of the as-prepared samples was observed by SEM (JSM-6390LV, JEOL) and TEM (JEOLJEM-2100, JEOL). Specific surface area and pore size distribution were calculated using the Brunauer–Emmett–Teller (BET) and density-functional theory (DFT) methods. The crystal structure of the as-prepared samples was collected by XRD (Bruker, Birrika, MS, USA). The bonding states and elemental composition were measured by FT-IR spectroscopy (Nicolet Nexus 470) and XPS (PHI5300/ESCA, Lafayette, LA, USA).

### 2.4. Electrochemical Measurements

The electrochemical performances of the as-prepared materials were evaluated in a 3 M KOH aqueous electrolyte by using the symmetrical two-electrode system. The as-prepared samples (85 wt.%), carbon black (10 wt.%), and polytetrafluoroethylene (5 wt.%) were mixed to prepare the electrode materials. CV, GCD, cycling performance, and leakage currents were tested on an electrochemical workstation (CSCT, Arbin, TX, USA). The single specific capacitance (F/g) was calculated with the following equation [32],

(1) $$C_{m}=\frac{2I\times \Delta t}{\Delta V\times m}$$

where *I* (A), Δ*t* (s), *m* (g), and Δ*V* (V) are the response current, the discharge time, the mass loading of the active materials in a single electrode, and the working potential, respectively.

## 3. Results and Discussion

**Figure 1:**
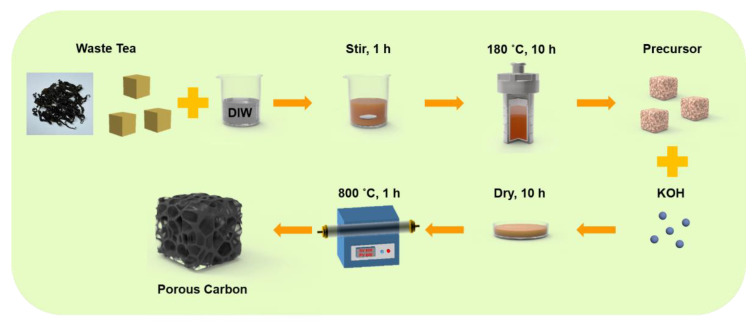
Schematic of the sequential preparation process of the tea-waste-based activated carbon materials.

**Figure 2:**
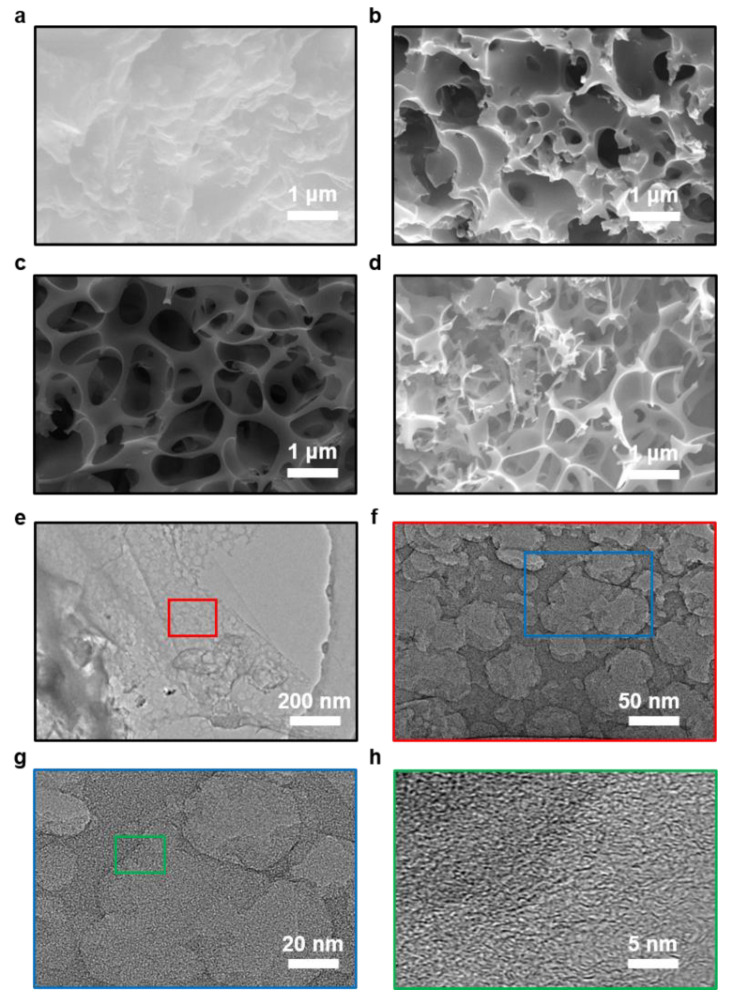
SEM images of the porous carbon electrodes: (**a**) TAC.; (**b**) TAC2; (**c**) TAC3; (**d**) TAC4; (**e**–**h**) TEM images of TAC3.

The schematic of the preparation process of the tea-waste-based activated carbon materials is shown in Figure 1. The morphology of the as-prepared samples was measured by SEM and TEM in Figure 2. The SEM image of TAC (Figure 2a) presents the relatively rough surface, which can facilitate the penetration of the KOH activation agent, contributing to the formation of the pores [33]. As shown in Figure 2b, the TAC2 starts to appear with some pores on the surface. Following the continuous increase in the KOH amount (i.e., TAC3), the randomly interconnected three-dimensional structure with different pore sizes is developed in Figure 2c. When the ratio increases to 4, the TAC4 exhibits a highly etched morphology with the fragmented structure (Figure 2d). These results suggested that the employed optimum KOH had an important effect on the morphology of the activated carbons. It is worth noting that the abundant pores of TAC3 may act as potential reservoirs to store the electrolyte and supply continuous energy. Moreover, the hierarchical structure can shorten the passway of the charge transfer and decrease the resistance of the ion diffusion to expect enhanced electrochemical performances. As presented in Figure 2e–g, TEM images of the TAC3 present a porous morphological configuration, which is in accordance with the result of the SEM images. Meanwhile, the high-resolution TEM image (Figure 2h) also reveals the amorphous nanostructures with a lower degree of ordering in the ACs [34,35].

**Figure 3:**
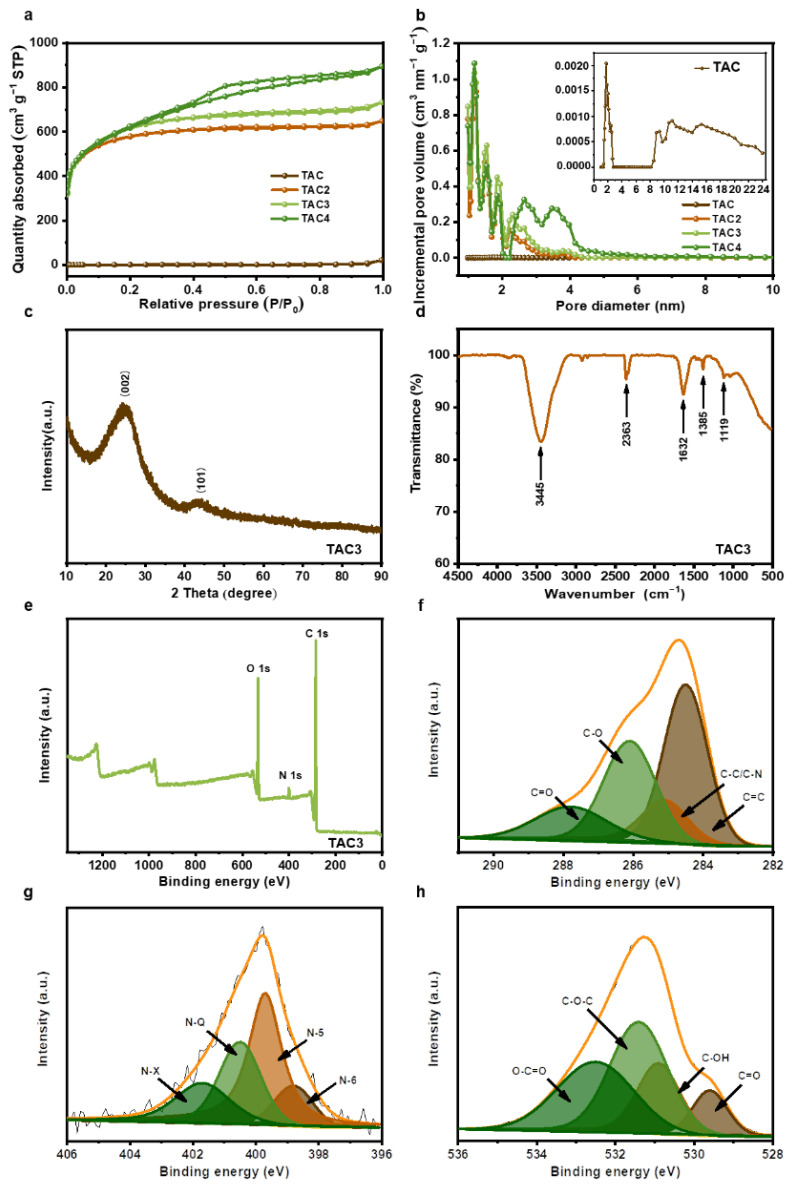
N_2_ adsorption–desorption isotherms (**a**) and pore size distributions (**b**) of tea-waste-based activated carbons, XRD pattern (**c**), FT-IR analysis (**d**), XPS survey spectra (**e**), and high-resolution C 1s (**f**), N 1s (**g**), and O 1s spectra (**h**) of TAC3.

The nitrogen adsorption/desorption was tested to investigate the surface areas and the related pore structures. As shown in Figure 3a, the isotherm of the TAC basically coincides with the line of Y = 0, and the adsorption amount was low in the structure, revealing the pore structure of the hydrothermal carbon was extremely underdeveloped. The isotherms of TAC2 and TAC3 are identified as I type, according to the IUPAC classification, in which the adsorption capacity increases rapidly with the increase in relative pressure when *P*/*P*_0_ < 0.1; the adsorption plateaus appear at a relative pressure between 0.1–0.4, indicating TAC2 and TAC3 are predominantly microporous structures. The adsorption/desorption isotherm of TAC4 exhibits a combined I/IV type [36,37], with a steep N_2_ uptake at *P*/*P*_0_ < 0.1 and a typical H4 hysteresis loop at *P*/*P*_0_ > 0.4, manifesting that TAC4 possesses micropores and mesopores. The pore size distribution curves are shown in Figure 3b, in which the pore sizes of TAC2, TAC3, and TAC4 were mainly distributed in ~1–5 nm and included both micropores (~1–2 nm) and mesopores (~2–5 nm). The pore size distribution of TAC (the inset of Figure 3b) consists of limited micropores and mesopores; the corresponding textural characteristics are summarized in Table 1. The TAC displays extremely low porosity with a specific surface area of 5 m^2^ g^−1^ and a pore volume of 0.034 cm^3^ g^−1^, which are similar to those of products derived from other types of biowaste [32,38,39]. By increasing the KOH agent, the specific surface area notably increases from 2143 m^2^ g^−1^ to 2235 m^2^ g^−1^ and then decreases to 2206 m^2^ g^−1^. In addition, mesoporosity gradually increases from 20.7% to 63.2% with the increased level of the activating KOH amount. This is because the KOH activation is divided into two parts: micropore formation (pore creation) and micropore expansion (pore expansion) [40]. While micropore features were induced upon K evaporation following infiltration, the existing micropores were expanded into mesopores by the gasification of carbonate (CO_3_^2−^) in K_2_CO_3_ [41]. However, when the ratio of KOH/TAC increases to 3, the micropores enlarge into mesopores, owing to excessive KOH etching [42]. The KOH activation mechanism is shown in the following equation [26,41]:

(2) $$6\mathrm{KOH}+2C\to 2K+3H_{2}+2K_{2}{\mathrm{CO}}_{3}$$

The large specific surface area with porous structures provides abundant active sites to accelerate the electron transfer and the ion diffusion that drives an improved electrochemical performance of the activated carbons. The microstructure of the TAC3 was readily measured by XRD. As a result of the XRD pattern in Figure 3c, the broad diffraction peak, at approximately 2*θ* = 25°, and the weak diffraction peak, centered at 43°, corresponded to the (002) crystal planes of the turbostratic carbon and the (101) diffraction of graphitic carbon, respectively, indicating the TAC3 belongs to the amorphous carbon with the disordered nature and low degree of graphitization [43,44], which is in agreement with the TEM results. Subsequently, the surface chemistries were characterized by FT-IR in the range of 4500–500 cm^−1^, as shown in Figure 3d. The broadband, at 3445 cm^−1^, and the weak peak, at 2363 cm^−1^, can be attributed to an O–H stretching and hydrogen-bonded or ionized compound structure [45]. The peaks at 1632 cm^−1^ are associated with the C=C/C=O stretching vibration [46,47]. The peaks at approximately 1385 and 1119 cm^−1^ indicate the vibration modes of the C-N and C-O-C groups [48,49]. These results revealed that the as-prepared carbons possessed a variety of surface functional groups, which were further evaluated by XPS spectroscopy. The XPS spectrum shows that TAC3 is mainly composed of carbon, nitrogen, and oxygen elements (Figure 3e). As presented in Figure 3f, the C 1s spectrum is divided into four peaks by Gaussian fitting, located at 284.5, 285.1, 286.1, and 287.8 eV, which are ascribed to the bonds of C=C, C-C/C-N, C-O, and C=O, respectively [17]. The high-resolution N 1s spectrum is deconvoluted into four peaks at 398.8, 399.7, 400.5, and 401.7 eV, are assigned to pyridinic-N (N-6), pyridone-N or pyrrolic-N (N-5), quaternary-N (N-Q), and pyridine N-oxide (N-X) as illustrated in Figure 3g [50,51]. The O 1s XPS spectrum is fitted into four peaks at approximately 531.3, 532.5, 533.2, and 533.9 eV, corresponding to the oxygen-containing groups C=O, C-O, C-O-C, and O-C=O, respectively, as presented in Figure 3h [17,52]. The above set of results suggests that TAC3 has various O-containing and N-containing groups on the surface. Importantly, these functional groups can effectively improve the hydrophilicity and wettability of activated carbon, thus making the surface accessible to aqueous electrolytes and capable of excellent electrochemical performances [53,54,55].

**Figure 4:**
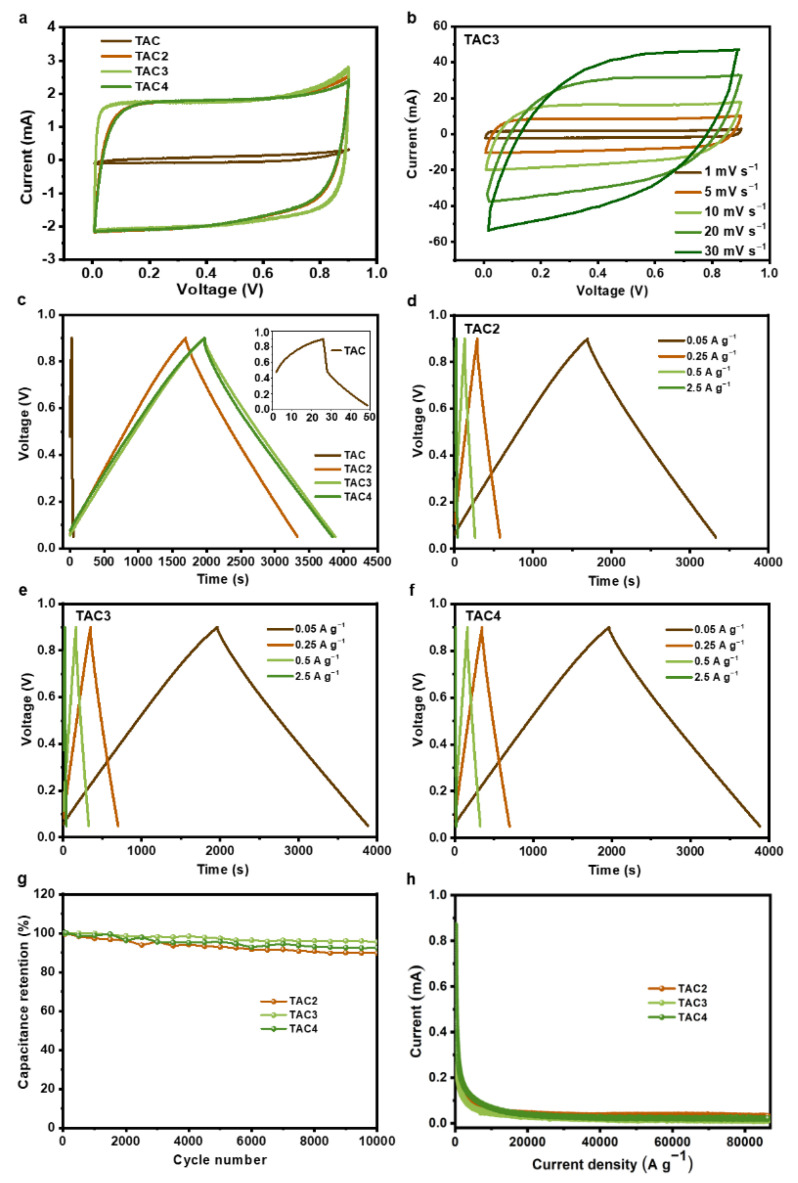
CV curves of the as-prepared samples at the scan rate of 1 mV s^−1^ (**a**); CV curves of TAC3 at different scan rates (**b**); GCD curves of all the samples at 0.05 A g^−1^ (**c**); GCD curves of TAC2 (**d**), TAC3 (**e**), and TAC4 (**f**) at different current densities; cycling performance under 1000 cycles of tea-waste-based activated carbons (**g**); and the leakage current curves of tea-waste-based activated carbons (**h**).

The as-prepared samples were tested as electrodes for the symmetric supercapacitors in a pair of electrode systems to analyze the electrochemical performances. Figure 4a presents the CV curves of the prepared samples at 1 mV s^−1^, in which the TAC electrode displayed a limited performance. In contrast, the CV curves from the TAC2, TAC3, and TAC4 electrodes represent quasi-rectangle shapes, demonstrating ideal capacitive behavior. Notably, the optimized capacitance was achieved from the TAC3 electrode. Moreover, as shown in Figure 4b, TAC3 retains an initial performance trend even at a high scan rate of 30 mV s^−1^, manifesting outstanding reversibility. Figure 4c illustrates the GCD curves of the tested samples at 0.05 A g^−1^, where the as-prepared TAC electrode only shows an evident initial voltage loss (i.e., IR drop). Meanwhile, the linear and nearly symmetric charge/discharge curves for the TAC2, TAC3, and TAC4 electrodes were collected without an obvious IR drop. Compared to other electrodes, the TAC3 electrode shows the longest discharge times that agreed well with the CV measurements. Within the specific survey of the GCD curves for the TAC2, TAC3, and TAC4 electrodes at various current densities, we found that the linear and symmetrical curves are also retained even at 2.5 A g^−1^, as shown in Figure 4d–f. Table 2 summarizes each capacitance of the prepared electrodes at the different current densities from 0.05 to 2.5 A g^−1^. The specific capacitance of TAC3 at 0.05 A g^−1^ reaches 256 F g^−1^, holding a high capacitance (221 F g^−1^) even at 2.5 A g^−1^ (~86.3% of the initial capacitance), whereas the TAC2 and TAC4 exhibit only ~79.8% and 66.9% capacitance retention, respectively. Obviously, the TAC3 electrode showed an excellent rate performance and high capacitance retention, which are crucial for high-performance supercapacitors. Additionally, long-term cyclic stability is an important parameter in investigating the electrochemical performances of supercapacitors. As measured in Figure 4e, the TAC3 maintains 95.4% after 10,000 cycles at 50 mA g^−1^, indicating a stable cycling performance compared to the other TAC2 and TAC4 electrodes. Additionally, when scanning the leakage current of electrodes for each sample, as shown in Figure 4f, the curves were overlapped with the values of 0.022, 0.014, and 0.031 mA for TAC2, TAC3, and TAC4 electrodes, respectively. The lowest leakage current value in the TAC3 electrode means the highest stability among the other samples and is important for practical applications as small as possible. Furthermore, the specific capacitance of TAC3 is compared with those of tea-waste-based carbon materials and other porous carbon materials, as listed in Table 3. It is noteworthy to mention that the competitive capacitance of TAC3 indicates that tea waste is a potential candidate as the electrode for high-performance supercapacitors.

**Figure 5:**
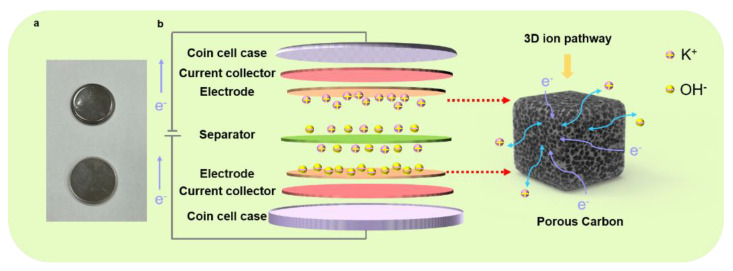
Digital image of the coin cell (**a**) and the schematic of the charging/discharging process of the symmetric supercapacitor (**b**).

As presented in Figure 5, we fabricated a coin-cell-type supercapacitor for a viable format to understand the charging/discharging process of the symmetric supercapacitors; the actual digital image of the coin cell shows a basic structure, and a schematic illustrates the charging/discharging process of the symmetric supercapacitors. In our experimental scheme, by designing the interconnected pores in the structured electrode, we postulate that the fluently transferred charges can be expected, and the ions can be freely diffused in the electrolyte. Based on the optimized condition for the supercapacitor electrode (i.e., TAC3), the excellent electrochemical performances can be attributed to the following reasons. Firstly, the TAC3 electrode yielded a high surface area with a value of 2235 m^2^ g^−1^, providing rich active sites and bringing a relatively large capacity during the charging/discharging process. Second, copious oxygen-containing functional groups at the surface of TAC3 were derived during the manufacturing process, exhibiting hydrophilicity and wettability, which is advantageous in contact conditions where electrolyte ions are easily accessible with the porous electrode. This characteristic feature in surface chemistry results in enhanced electrochemical performances. Furthermore, the interconnected frameworks in the porous electrode offer the intrinsic passways rapid ion diffusion and charge transfer, promoting cycling stability and reversibility. Within this combinatorial condition, our experimental results demonstrate an outstanding electrochemical performance of the tea-waste-based electrodes. Thus, we believe that our present study may provide an extremely simple hydrothermal method in designing practical electrode materials for supercapacitor applications by utilizing low-cost and eco-friendly biowastes as the raw materials combined with KOH activation.

## 4. Conclusions

In summary, we developed a simple process to produce porous carbons derived from tea waste using hydrothermal treatment with KOH activation. During the process, KOH, as an activating agent, reacts with carbon to create and expand pores. In this work, we found that the amount of KOH has a crucial impact on the microstructure and electrochemical performances. Among the porous carbons, TAC3, with an interconnected framework, presents the highest specific surface area of 2235 cm^3^ g^−1^ and high hydrophilicity. When used as the electrode for the supercapacitor, TAC3 delivers an excellent specific capacitance (256 F g^−1^) at 0.05 A g^−1^ and retains 221 F g^−1^ at 2.5 A g^−1^. Meanwhile, the capacitance maintains 95.4% after 10,000 cycles, and the leakage current is only 0.014 mA. These results demonstrate that tea waste is a promising candidate to synthesize electrode materials for high-performance supercapacitors. Our work provides an effective and feasible approach to recycling eco-friendly and low-cost biowastes and increasing value-added utilization in the field of energy storage, which is fitted with the demands of reducing pollution and carbon emissions [62,63].

## Figures and Tables

**Table 1:** Textural characteristics of the activated carbons.

Samples	*S*_BET_ m^2^ g^−1^	*V*_total_ cm^3^ g^−1^	*V*_mic_ cm^3^ g^−1^	*V*_mes_ cm^3^ g^−1^	*V*_mes_/*V*_total_ %
TAC	5	0.034	0.002	0.032	94.1
TAC2	2143	1.006	0.798	0.208	20.7
TAC3	2235	1.135	0.796	0.339	29.9
TAC4	2206	1.387	0.511	0.836	63.2

**Table 2:** The specific capacitances of the activated carbons at different current densities.

Samples	Specific Capacitance (F g^−1^)				Capacitance Retention *
	0.05 A g^−1^	0.25 A g^−1^	0.5 A g^−1^	2.5 A g^−1^	
TAC	5	-	-	-	-
TAC2	218	201	193	174	79.8%
TAC3	256	238	232	221	86.3%
TAC4	242	201	200	162	66.9%

Note: * Capacitance retention is the capacitance at 2.5 A g^−1^ divided by the capacitance at 0.05 A g^−1^.

**Table 3:** The specific capacitances of the as-prepared carbon compared with those of the tea-waste-based carbon materials and other porous carbon materials.

Active Materials	Current Density (A g^−1^)	Specific Capacitance (F g^−1^)	References
tea factory waste	2	155	[12]
green tea waste-derived ultrathin mesoporous graphitic carbon nanoflakes	0.5	162	[26]
tea-waste-based activated carbon	0.1	140	[27]
tea-waste-based, multi-hierarchical porous carbon	1	291.2	[56]
microporous and mesoporous activated carbons produced from tea waste	1.5 mA cm^2^	203	[28]
activated carbons derived from tea leaf waste	1	330	[24]
hierarchical porous carbon with multi-heteroatom co-doping from tea waste	0.5	170	[57]
activated biomass carbon from tea leaves	0.5	131.95	[58]
hierarchically porous carbon nanosheets from coffee grounds waste	0.5	129	[59]
biowaste lemon-peel-derived carbon	0.2	106	[20]
sesame husk-based activated carbon	2.5	235	[19]
MnO_X_-modified corrugated carton-derived hierarchical porous carbon	2.5	279	[17]
activated carbon derived from anaerobic digester residues	1	184	[48]
hierarchical N-doped porous carbon nanosheet material from soybean milk	0.5	149	[26]
heteroatom-doped porous carbon sheets derived from protein-rich wheat gluten	0.5	350	[60]
activated carbon derived from rotten carrot	10 mHz	135.5	[61]
porous carbons derived from tea waste	2.5	221	This work

## Data Availability

The data presented in this study are available on request from the corresponding authors.
